# A Population-Based Study of Pre-Existing Health Conditions in Traumatic Brain Injury

**DOI:** 10.1089/neur.2020.0065

**Published:** 2021-06-09

**Authors:** Kristine C. Dell, Emily C. Grossner, Jason Staph, Philip Schatz, Frank G. Hillary

**Affiliations:** ^1^ Department of Psychology, The Pennsylvania State University, University Park, Pennsylvania, USA.; ^2^ Social and Life and Engineering Sciences Imaging Center, The Pennsylvania State University, University Park, Pennsylvania, USA.; ^3^ Department of Psychology, Saint Joseph's University, Philadelphia, Pennsylvania, USA.; ^4^ Department of Neurology, Hershey Medical Center, Hershey, Pennsylvania, USA.

**Keywords:** cluster analysis, pre-existing conditions, traumatic brain injury

## Abstract

Health factors impacting both the occurrence of, and recovery from traumatic brain injury (TBI) vary in complexity, and present genuine challenges to researchers and healthcare professionals seeking to characterize injury consequences and determine prognosis. However, attempts to clarify causal links between injury characteristics and clinical outcomes (including mortality) often compel researchers to exclude pre-existing health conditions (PECs) in their samples, including psychiatric history, medication usage, and other comorbid conditions. In this pre-registered population-based study (total starting *n* = 939,123 patients), we examined trends in PEC incidence over 22 years in the state of Pennsylvania (1997–2019) in individuals sustaining TBI (*n* = 169,452) and individuals with orthopedic injury (*n* = 87,637). The goal was to determine how PECs interact with age and injury severity to influence short-term outcomes. A further goal was to determine whether number of PECs, or specific PEC clusters contributed to worse outcomes within the TBI cohort, compared with orthopedic injury alone. Primary findings indicate that PECs significantly influenced mortality within the TBI cohort; patients having four or more PECs were associated with approximately a two times greater likelihood of dying in acute care (odds ratio [OR] 1.9). Additionally, cluster analyses revealed four distinct PEC clusters that are age and TBI severity dependent. Overall, the likelihood of zero PECs hovers at ∼25%, which is critical to consider in TBI outcomes work and could potentially contribute to the challenges facing intervention science with regard to reproducibility of findings.

## Introduction

Despite the dense literatures examining the role of demographic and injury related/severity factors that influence early recovery from traumatic brain injury (TBI),^1–8^ pre-existing health conditions (PECs) are relatively under-studied outcomes. Health conditions co-occurring with injury increase as a function of advancing age, and contribute to variability in both post-injury trajectories as well as heterogeneous factors involved in recovery.^9–14^ Emerging research has demonstrated that comorbidities can complicate functional recovery and symptom resolution. However, these studies frequently focus on select (notably psychiatric) PECs, or examine the contribution of TBI to exacerbated systemic disorders or illness rather than the opposing directionality.^15,16^ Similarly, incongruity in the terminology utilized to discuss PECs, combined with diversity in identification and classification methods of these conditions (self-report, extraction from medical records, semi-structured interview), can also complicate our ability to reconcile findings from existing studies. For our purposes, the term PEC is used to signify conditions or disorders present, self-reported, or otherwise diagnosed before the time of injury. This is in contrast to “nosocomial conditions,” which are healthcare-associated conditions originating during or as a result of a hospital stay,^17–19^ and “comorbid” conditions such as substance abuse and psychiatric response to injury that are the focus months to years after the injury occurred.^13,14,20–24^

The Centers for Disease Control and Prevention's (CDC's) National Center for Chronic Disease Prevention and Health Promotion (NCCDPHP) estimates that 6 out of every 10 adults (18 years or older) in the United States have at least one chronic disease, and 4 out of 10 adults have at least two chronic conditions.^25^ Over the last 10 years, cardiac conditions, psychiatric conditions, diabetes, respiratory disorders, and musculoskeletal conditions have remained stable as the most frequent chronic conditions.^26^ Further, the NCCDPHP denotes heart disease, dementias, diabetes, arthritis, and cancer as the leading contributors of disability, death, and healthcare costs in the United States, with age conferring increased risk of these disease conditions.^25^

Understanding the influence of the increased disease burden introduced by the development and progression of these PECs is critical across all severities of TBI.^15,16,27^ In addition, the impact of PECs does not appear limited to the acute period following TBI. Patients with moderate-to-severe TBI (msTBI) with a greater total disease burden reported reduced levels of functioning and life satisfaction up to 10 years following injury.^28^ Together, these studies add to the growing literature examining the impacts of both psychiatric^1,10,13,14,20–23,29^ and other medical conditions^24,30–34^ on outcomes following TBI. Such multi-morbidity adds complexity to the care required for these TBI patients in comparison to individuals with singular or no reported PECs and provides evidence for a systemic disease burden on the individual. Similarly, clinical care focused on a single diagnosis may not accurately depict the patient's comprehensive clinical picture or rehabilitative needs.^15,24,35–37^

In patients older than age 50 years with msTBI, hypertension emerged as a primary PEC and along with related medical complications, predicted hospitalization at 1 year post-injury.^27^ In elderly patients with TBI, not only are PECs associated with worse outcomes, but morbidity is more highly linked to PEC complications than complications from the injury.^27,38^ In a cohort of elderly patients with TBI following treatment in a neurosurgical department, patients with comorbid cardiac, pulmonary, or renal conditions and malignancy were almost three times more likely to die or enter a vegetative state post-injury.^39^ Related work has shown that patients age 50 years or older are significantly more likely to have diabetes, elevated cholesterol, osteoarthritis, and hypertension in comparison with individuals under the age of 50 who sustain TBI.^40^

Although it may be difficult to directly link the onset of these conditions post-injury as a result of TBI or aging, what does emerge is evidence for reciprocal impacts of central and peripheral nervous system functioning,^19,41–43^ and how this interplay may confer risk of pathological aging in the form of subsequent injury, disease progression, or exacerbated recovery.^30,44–46^ Studying TBI without understanding or incorporating PECs not only has broad implications for the reproducibility of findings,^47^ but also risks undermining the detection of important pre-injury and recovery phenotypes.^28,48^ Similarly, it may also play a role in the near complete failure in advancing phase 2 clinical trials for brain injury interventions.^41,49,50^

### Study goal

Given the importance of understanding how the presence of PECs can contribute to the heterogeneity of TBI recovery trajectories, we conducted a population-based study to identify the base rates of PECs in TBI, to examine the influence of PECs on TBI outcome, and to confirm the previously identified prevalence of PECs in individuals with TBI. Although we examined all cases of TBI during those years, we focused on moderate and severe TBI in analyses to determine the interaction between PEC and injury severity in predicting mortality and functional outcomes. We also sought to determine if PECs interact specifically with moderate and severe TBI compared with orthopedic injury to confer specific risk of prolonged hospital stay and diminished functional status at discharge.

### Hypotheses

#### Pre-registered hypotheses

1. Mortality rate and days spent in the hospital will be positively correlated with the quantity of PECs.
2. Patients with TBI with a greater number of PECs will perform worse on measures of independence, compared with patients with TBI with fewer PECs.
3. PEC subtypes will be associated with poor outcomes in elderly patients with TBI.

#### Exploratory questions (all developed before data analysis)

1. What can the results of K-modes clustering demonstrate regarding specific characteristics of patients with TBI presenting with a higher number of PECs?
2. Does TBI (as opposed to trauma-only) select for certain PECs when examining all patients?
3. Does the frequency of PEC profiles change over the lifespan?

## Methods

Beginning in October 1986, the Pennsylvania Trauma Systems Foundation (PTSF) initiated a trauma registry (Pennsylvania Trauma Outcomes Study, or PTOS) to receive data from participating hospitals, in an effort to evaluate patient outcomes through research and education across comparable centers state-wide. At the authors' request, the PTSF provided de-identified data collected from 1986 to June 2019 comprising two approximately equal-sized patient cohorts (total *n* = 939,123 cases): trauma patients entering emergency departments (EDs) with a documented TBI (*n* = 476,006), and a second group of patients without a documented TBI at admission (orthopedic control group, *n* = 463,117). The TBI and orthopedic injury cases were selected according to codes specific to TBI (*International Classification of Diseases*, 9th Revision [ICD9] code prefixes: 800–806, 850–854, 950, 951, 952; ICD10 codes: S02, S04, S06, S07, S12, S14.0–S14.3, S22.0–S22.089, S32.0–S32.3, S34.0–S34.2, S24.0–S24.2). When the PTSF provided the data, they included the binary variable “TBI” denoting patients with a diagnosis code from the requested code range, whereas patients with orthopedic injury were those without any of the listed codes. Due to the size of the raw dataset (2.1 GB, 1.2 billion cells of data), data cleaning and analysis required a multi-step process implemented through several software applications.

For the framework outlined below, version control and data provenance were performed through git^51^ and git annex.^52^ Data were received from the PTSF in two CSV formatted files, one for each requested cohort (TBI and orthopedic injury). Simple CSV validity checks were performed on the data with csv-kit,^53^ then the two cohort files were converted to TSV to increase speed and efficiency of use, and then were combined. Initial evaluation of the data was performed via a Docker^54^ based Elasticsearch^55^ and Kibana^56^ configuration, using Logstash^57^ to consume the raw data, and to index the resulting records to Elasticsearch.^55^ Kibana^56^ was used as an explorational tool for the data and data cleaning, and normalization was then implemented using a combination of Python^58^ and R.^59^ All codes used to alter the data were tracked in git,^51^ with original source data all subsequent revised versions tracked through git annex.^52^ Docker^54^ compose configuration files were created to automate the use of the Elasticsearch^55^ and Kibana^56^ tools to provide consistency in the software environment across all computing resources.

To facilitate further processing and reporting on the data, sub-datasets were created through selection of limited numbers of columns, selection of records based on column values (post-cleaning), and normalization of the data within columns (converting “<UNK>” and “<n/a>” to either null values, or the appropriate integer value for the column in which they were discovered). For data fields of interest, all unique values present in the data were collated, and compared with the “2017 Pennsylvania Trauma Systems Foundation Operational Manual for the Database Collection System”^60^ for compliance with data entry guidelines. Data cleaning steps were applied where the unique values differed from the operational manual's allowed values, in the following ways:

1. “<n/a>,” “<UNK>,” and “–,” were set to “–1,” −2,” and “–3,” respectively, in integer fields.
2. “<UNK>” and “<n/a>” were replaced with an empty string in text fields.
3. Date and Time fields were converted from several conventions to conform to ISO 8601 format. If the date or time was “<UNK>” or “<n/a>,” it was converted to an empty string.

**FIG. 1.**
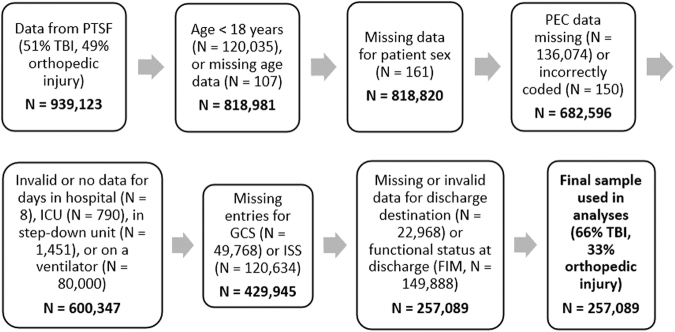
List-wise deletion of cases with missing data during data cleaning. Each box identifies reasons cases were moved to create the final sample used for analysis. FIM, functional independence measure; GCS, Glasgow Coma Scale; ICU, intensive care unit; ISS, injury severity score; PEC, pre-existing condition; PTSF, Pennsylvania Trauma Systems Foundation; TBI, traumatic brain injury.

After list-wise deletion of cases with missing data (Fig. 1), 257,089 cases were available for use in the present analyses dating from 1997 until 2019.

### K-modes clustering analysis to establish PEC profiles

**FIG. 2.**
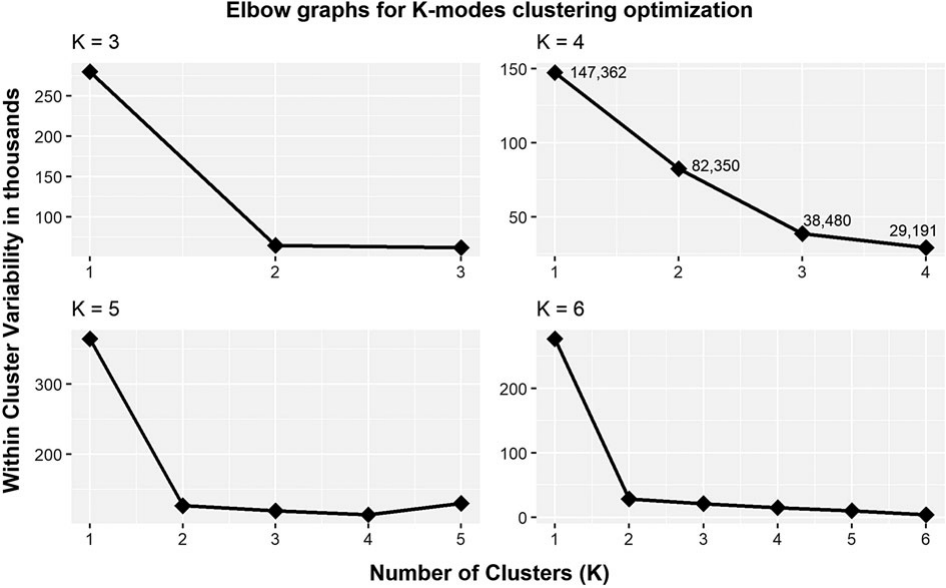
Elbow plots for K-modes clustering analysis, plotting within cluster variability values across numbers of clusters set to K = 3, 4, 5, or 6. Visual inspection of these four plots confirmed K = 4 as the optimal number of clusters.

Given that the PTOS permits coding of up to 10 distinct PEC entries, we elected to include all patients (*n* = 257,089), regardless of the presence of TBI or injury severity together, to implement a data-driven approach (K-modes clustering algorithm using the *klaR*^61^ package in R)^59^ to aggregate clusters of PEC profiles. Only a binary matrix representing the presence or absence of each PEC for each patient was included in the K-modes analysis; demographic or injury variables were not included. We then sought to validate the optimal number of clusters in the manner consistent with one of the traditional validation methods of examining elbow plots of the within-cluster sum of squares (wss) implemented in K-means clustering,^62^ given K-modes has been presented as an extended application of the original K-means algorithm.^63,64^ Within the K-modes function, we set an initial seed randomly at the value of (12345), estimated an initial number of clusters (K) equal to 4, and requested a maximum of 10 iterations. To confirm the optimal number of clusters, we repeated these steps, with the exception of changing the value of K to 3, 5, and then 6, and subsequently plotted the wss values returned by the K-modes function for each of our runs. The resulting plots are those presented in Figure 2. 

A comprehensive list of the 19 PEC categories, which the PTOS defines as “pre-existing comorbid factors present before patient arrival at the Emergency Department (ED)/hospital,” are provided in Supplementary Table S1 and the PTOS data manual.^60^ Of note, several of the PECs align with and use the same definition as the National Trauma Data Bank (NTDB) data dictionary.^60^ Further, during the process of data cleaning, the PEC category of Pregnancy was dropped from the dataset to remove bias in PEC count for female trauma patients represented in the database, leaving a total of 18 PEC categories for the current analyses across 43 different participating trauma centers throughout the state.

After filtering out mild TBI (mTBI) cases (Glasgow Coma Scale [GCS] score 12–15; *n* = 150,721) and orthopedic injury cases (*n* = 87,633), we conducted logistic regression with total count of PECs as the independent variable for hypotheses examining the impact of PECs on mortality in patients with msTBI (*n* = 18,729), using the *glm* function in R.^59^ To ensure comparable cell sizes for the logistic regression, we collapsed PEC quantities 6 through 10 into one group with a PEC count value of 6 or greater (≥6). For the remaining hypotheses regarding functional independence (FIM) and other hospital outcomes variables (days spent in the hospital, days in the intensive care unit [ICU]), we conducted linear regression on each of the three dependent variables with either PEC count or K-modes cluster assignment as the independent variables.

## Results

Demographics for all subjects are presented in Table 1. Due to the high degree of missingness for the Ethnicity variable specifically (30–90%), it was not feasible to make reliable calculations for participants of Hispanic/Latino descent.

**Table 1A.** Demographics of Patients with and without Recorded PEC Data Prior to Data Cleaning

Data	Race^a^	% of n	$\bar{M}$ age in years (SD)	Male	Discharged alive	Orthopedic
Adult patients with PEC data available at project start:	Caucasian	78%	55.3 (22.8)	58%	95%	46%
*N* = 796,756	African-American	15%	40.7 (18.5)	73%	92%	59%
52% TBI	Asian	0.8%	49.7 (21.2)	58%	94%	44%
Median GCS score = 15	Other	2.3%	42.2 (19.2)	72%	94%	49%
45% have zero PECs	Unknown	3.2%	44.1 (20.2)	72%	94%	52%
	No data	0.2%	45.6 (20.9)	65%	94%	49%
Adult patients w/out PEC data available at project start^b^:	Caucasian	67%	45.4 (21.1)	67%	77%	41%
*N* = 39,771	African-American	25%	34.9 (16.5)	84%	55%	57%
48% TBI	Asian	1.3%	40.6 (16.8)	74%	68%	42%
Median GCS = 3	Other	3.2%	36.2 (16.3)	83%	73%	42%
	No data	3.3%	40.0 (21.4)	85%	47%	47%

^a^ Race and ethnicity data were not available for all patients. Due to the high degree of missingness for the Ethnicity variable, percentages of patients of Latino/Hispanic origin could not be reliably calculated.

^b^ Patients without PEC data excluded from further analysis.

GCS, Glasgow Coma Scale; PEC, pre-exisiting health condition; SD, standard deviation; TBI, traumatic brain injury.

**Table 1B.** Sub-Sample Demographics following Data Cleaning

Final data samples	Race^a^	% of n	$\bar{M}$ age in years (SD)	Male	Discharged alive	Most frequent discharge destination
Mild TBI:	Caucasian	84%	56.2 (22.8)	59%	100%	Home
*N* = 150,721	African-American	9%	43.3 (18.6)	73%	100%	Home
27% have zero PECs	Asian	0.9%	51.7 (21.4)	57%	100%	Home
	Other	2.1%	43.7 (19.7)	70%	100%	Home
	No data	4%	45.2 (20.2)	71%	100%	Home
Moderate TBI:	Caucasian	76%	54.0 (23.5)	62%	100%	Home, rehab facility
*N* = 5839	African-American	14%	43.1 (18.1)	78%	100%	Home, rehab facility
26% have zero PECs	Asian	1.6%	50.6 (23.2)	62%	100%	Home, rehab facility
	Other	3%	43.4 (20.1)	79%	100%	Home, rehab facility
	No data	5.4%	43.4 (20.1)	73%	100%	Home, rehab facility
Severe TBI:	Caucasian	82%	41.2 (18.8)	74%	100%	Rehab facility, LTC
*N* = 12,890	African-American	10%	39.8 (16.6)	83%	100%	Home, rehab facility^b^
38% have zero PECs	Asian	0.9%	42.6 (17.5)	60%	100%	Rehab facility, home
	Other	2.8%	37.0 (16.4)	80%	100%	Home, rehab facility
	No data	4.8%	38.0 (16.9)	80%	100%	Home, rehab facility^b^
Orthopedic Injury:	Caucasian	77%	54.2 (21.8)	60%	100%	Home
*N* = 87,633	African-American	16%	37.4 (16.6)	77%	100%	Home
30% have zero PECs	Asian	0.8%	46.9 (20.1)	61%	100%	Home
	Other	2.2%	40.2 (17.3)	74%	100%	Home
	No data	4%	40.3 (18.1)	75%	100%	Home

^a^ Race and ethnicity data were not available for all patients. Due to the high degree of missingness for the Ethnicity variable, percentages of patients of Latino/Hispanic origin could not be reliably calculated.

^b^ Discharge destinations were equal in frequency.

LTC, long term care facility; PEC, pre-existing health condition; SD, standard deviation; TBI, traumatic brain injury.

### Hypothesis 1: PECs will influence mortality and hospital outcomes

We analyzed mortality within a cohort of adult patients with msTBI (*n* = 50,574; discharged alive = 73%). Logistic regression revealed a significant overall effect of PEC quantity in predicting mortality (χ^2^ [6, *n* = 50,574] = 427.3, *p <* 0.001). Individual Wald's tests for each level of PEC confirmed each additional PEC significantly increased the odds of patients with msTBI being discharged dead in comparison with patients with msTBI admitted with zero PECs. When comparing patients with TBI without any PECs (odds ratio, [OR] = 0.28), the ORs for TBI cases with one or more diagnosed PEC were (from 1 to 6 PECs): 1.3, 1.5, 1.6, 1.9, 1.7, and 1.7, respectively. For example, individuals with 4 PECs were nearly twice (1.9 times) more likely to die compared with those with no PECs. Separate Wald's tests also confirmed that the differences in the coefficients for each additional level of PEC (0 versus 1, 1 versus 2, 2 versus 3, etc.) were all statistically significant, with the exception of the difference in coefficients for PEC quantities 5 and 6 (χ^2^ [1, *n* = 2328] = 0.032, *p* = 0.86).

### Hypothesis 2: Number of PECs will influence outcomes in patients with TBI

**FIG. 3.**
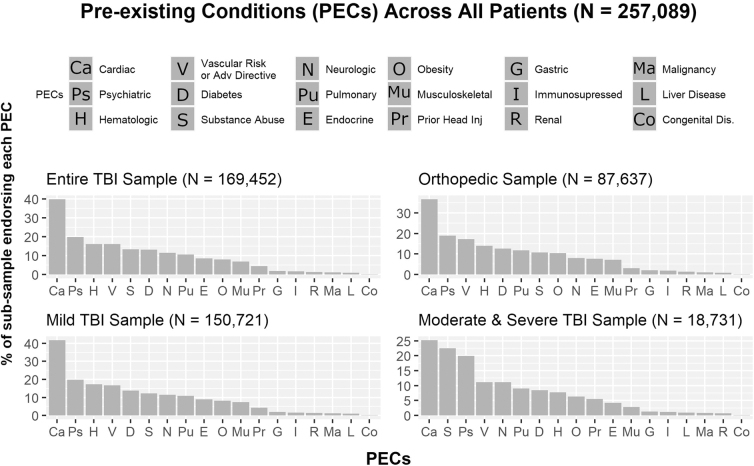
Relative frequency of distinct PECs. Note frequencies for the Entire TBI Sample, Mild TBI Sample, and Orthopedic Sample remain similar. The Moderate and Severe TBI Sample reveals higher frequencies for substance use (1 in approximately 4) compared with the other three frequency graphs (1 in approximately 7). PEC, pre-existing condition; TBI, traumatic brain injury.

The percentages of patients across injury sub-samples reported for each PEC category are presented in Figure 3. The PEC counts of patients with msTBI significantly predicted FIM scores (*F* [*1, 18729*] = 206.1, *p <* 0.001, η^2^ = 0.013). For each additional PEC, FIM scores were predicted to decrease by half a point (*b* = −0.5, *t* [18729] = −14.4, *p* < 0.001). Similarly, PEC counts of patients with msTBI significantly predicted total days spent in the ICU, (*F* [*1, 18729*] = 9.3, *p* = 0.002, η^2^ = 0.001). More specifically, each additional PEC is expected to decrease days spent in the ICU by 4.8 h, (*b* = −0.2, *t* [18729] = −3.0, *p* = 0.002). To examine the impact of the number of PECs on total days spent in the hospital, we collapsed patients with PEC counts of 3 or more into one group with an assigned PEC count value of 3. The total number of diagnosed PECs did not significantly impact the number of days patients with msTBI spent in the hospital, (*F* [*3, 18727*] = 1.64, *p* = 0.18, η^2^ < 0.001).

### Hypothesis 3: Examining how PECs combine to confer risk

**FIG. 4.**
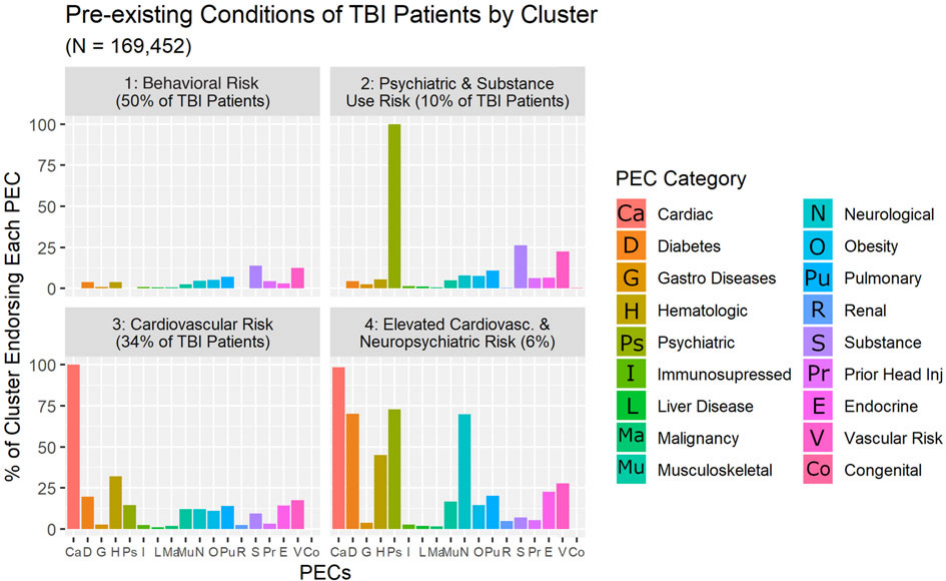
Results of the K-modes cluster analysis. Four clusters are represented in panels: 1) Behavioral Risk: which includes patients with no recorded PECs, and individuals whose primary diagnosed PECs were Substance Use Disorder and/or Current Smoker; 2) Psychiatric and Substance Use Risk: primary PEC diagnoses were Psychological or Personality Disorder, followed by Substance Use Disorder, and Current Smoker; 3) Cardiovascular Risk: patients with predominantly Cardiac Conditions, followed by Hematologic, Diabetes, and Vascular Risk PECs; 4) Elevated Cardiovascular and Neuropsychiatric Risk: patients primarily with Cardiac, Psychiatric, Diabetes, Neurological, and Vascular Risk conditions. PEC, pre-existing condition; TBI, traumatic brain injury.

Comparison of the four elbow plots generated by the various K-modes clustering runs confirmed four as the optimal number of PEC profiles (Fig. 2). We subsequently named these clusters based on their predominant PEC categories (Fig. 4). The four clusters that emerged were: 1) Behavioral Risk, which includes patients with zero recorded PECs (*n* = 6387), and individuals whose primary diagnosed PECs were Substance Use Disorder and/or Current Smoker (*n* = 5087); 2) Psychiatric and Substance Use Risk; 3) Cardiovascular Risk; and 4) Elevated Cardiovascular and Neuropsychiatric Risk, which consists of patients with a combination of three or more comorbid PEC diagnoses. Additional information regarding characteristic PEC sub-categories and demographics of these clusters is provided in Table 2 .

**Table 2.** Descriptive Characteristics by K-Modes Cluster of Patients with Moderate and Severe TBI

Cluster (*n*)	$\bar{M}$ age (SD) in years	Male	Caucasian^a^ (African-American)	Median hospital days	$\bar{M}$ GCS score on admission
Behavioral Risk (total *n* = 11,474), consisting of two subgroups					
1. No PECs reported (6,387, 56%)	33 (14.3)	78%	83% (11%)	10	5.6
	% of group endorsing trait		% only endorsing this trait		
No PECs reported		100%		100%		
2. PEC-positive (5087, 44%)	41 (17.4)	77%	81% (15%)	9	6.1	
	% of group endorsing trait		% only endorsing this trait			
Chronic alcohol abuse Substance use disorder	35%		23%			
	22%		15%			
Psychiatric and Substance Use Risk (2523)	39 (15.9)	65%	89% (9%)	10	6.1	
PEC sub-categories^b^	% of cluster endorsing trait		% only endorsing this trait			
Diagnosed psychiatric/Personality disorder Chronic alcohol abuse Substance use disorder Current smoker Diagnosed ADHD Respiratory disease or COPD	91%		31%			
	25%		0%			
	19%		0%			
	14%		0%			
	10%		4%			
	10%		0%			
Cardiovascular Risk (3903)	64 (17.2)	67%	87% (11%)	11	6.8	
PEC sub-categories	% of cluster endorsing trait		% only endorsing this trait			
Hypertension requiring medication Coronary artery disease Chronic alcohol abuse Diabetes mellitus Diagnosed psychiatric/Personality disorder Obesity Respiratory disease or COPD	89%		18%			
	22%		3%			
	17%		0%			
	16%		0%			
	15%		0%			
	10%		0%			
	14%		0%			
Elevated Cardiovascular and Neuropsychiatric Risk (831)	72 (15.7)	50%	89% (9%)	7	8.3	
PEC sub-categories	% of cluster endorsing trait		% only endorsing this trait			
Hypertension requiring medication Diagnosed psychiatric/Personality disorder Diabetes mellitus Dementia CVA/Hemiparesis (stroke with residual) Coronary artery disease Anticoagulant therapy Respiratory disease or COPD Seizures Functionally dependent health status Congestive heart failure Arthritis Obesity Chronic alcohol abuse History of cardiac surgery Advanced directive-limited care	87%		0%			
	71%		0%			
	66%		0%			
	49%^c^		0%			
	31%		0%			
	33%		0%			
	29%^d^		0%			
	19%		0%			
	14%		0%			
	14%		0%			
	14%		0%			
	11%		0%			
	11% 11%		0% 0%			
	10.5%		0% 0%			
	10%					

^a^ Race data were not available for all participants.

^b^ PEC sub-categories were those endorsed by at least 10% of the cluster.

^c^ Coding changes over the lifetime of the PTOS database collapsed previously separate codes for 1. Alzheimer's disease and 2. chronic dementia, into a new third code for Dementia. The percentage in this table is the sum of those three subcategories.

^d^ Coding collapsed previously separate codes for Anticoagulant therapy, Anti-platelet agents, and Pradaxa therapy in to one new code for Anticoagulant therapy. The percentage in this table is the sum of those four subcategories.

ADHD, attention deficit hyperactivity disorder; COPD, chronic obstructive pulmonary disease; CVD, cardiovascular disease; GCS, Glasgow Coma Scale; PEC, pre-exisiting health condition; PTOS, Pennsylvania Trauma Outcomes Study; SD, standard deviation; TBI, traumatic brain injury.

### Exploratory question 1: Characteristics of patients with increasing quantities of PECs

Examination of PEC counts (patients with msTBI reporting 0, 1 to 2, or 3 or more PECs) regardless of cluster membership reveals descriptive differences across the three groups with respect to demographics, but not hospital outcomes variables. Increasing PEC counts are positively associated with age and the proportion of female patients, but not with increasing stays in the ICU or total hospital days. However, the average hospital lengths of stay and days spent in the ICU were approximately 14 total days in the hospital and around 8 days in the ICU for all three PEC count groups.

### Exploratory question 2: Frequency of PECs within TBI and orthopedic injury

Comparison of the entire TBI sample with the orthopedic injury sample reveals similar frequencies of PEC categories across both groups, supporting the decision to include all patients regardless of injury status in the K-modes clustering algorithm. The four most frequently reported PECs categories for both orthopedic patients and patients with mTBI were cardiac, psychiatric, hematologic, and vascular risk (predominantly smoking) conditions. These same frequencies, however, did not hold for patients with msTBI, with cardiac, substance use, psychiatric, vascular risk (again predominantly smoking), and neurological conditions reported in the msTBI group.

### Exploratory question 3: Change in PECs over the lifespan

**FIG. 5.**
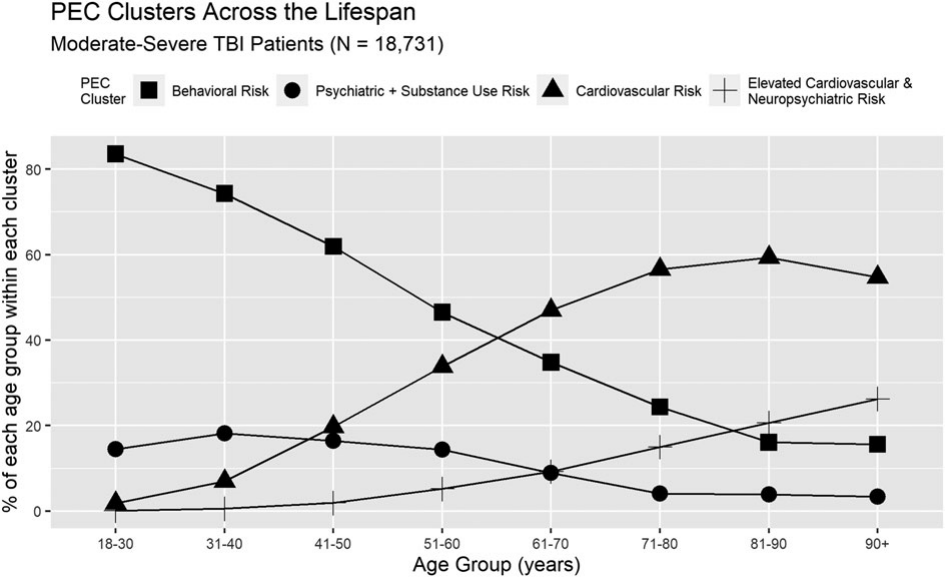
Patient age plays a role in membership to specific PEC clusters. The observed shifts appear most notable between the fifth and sixth decades of life, but can emerge as early as a patient's forties, and persist in to a patient's seventies. PEC, pre-existing condition; TBI, traumatic brain injury.

K-modes results demonstrate a clear demographic shift in the qualitative and quantitative characteristics (type and count) of the PEC clusters within msTBI (see Fig. 5). More specifically, the Behavioral Risk and Psychiatric and Substance Use Risk PEC profile groups are both younger on average than the Cardiovascular Risk and Elevated Cardiovascular and Neuropsychiatric Risk clusters. Shifts in the emergence of PEC cluster types are most evident when patients are in their fifties and sixties, but emerge as early as one's forties (Cardiovascular Risk cluster), and persist in to one's seventies (Elevated Cardiovascular + Neuropsychiatric Risk). Further, as the mean age of the PEC clusters begins to increase, so too do the proportions of female patients represented within each cluster.

To ensure equal cell sizes for the one-way analysis of variance (ANOVA) tests (Welch's *F*), we randomly sampled 500 cases per cluster from the total sub-sample of patients with msTBI (*n* = 18,731). Results revealed cluster group differences in FIM (*F* [3, 1108.5] = 51.4, *p* < 0.001) and lengths of stay both in the ICU (*F* [3, 1105.5] = 7.3, *p* < 0.001) as well as the hospital overall (*F* [3, 1099.9] = 5.7, *p* < 0.001). Post hoc tests with Bonferroni correction revealed mean differences in FIM scores between the Behavioral Risk cluster ($\bar{M}=14.9$) and the two oldest clusters: Cardiovascular Risk 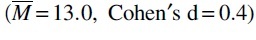 and Elevated Cardiovascular and Neuropsychiatric Risk ($\bar{M}=12.1,Cohen\prime sd=0.6;$ both *p* < 0.001), but no significant differences in mean FIM scores when compared with the Psychiatric and Substance Use Risk cluster 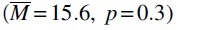. Similarly, the Psychiatric and Substance Use Risk cluster mean FIM score was also significantly higher than the two oldest clusters (both *p* < 0.001). Finally, mean FIM scores did not differ significantly between the Cardiovascular Risk and Elevated Cardiovascular and Neuropsychiatric Risk, *p* = 0.08.

With respect to total days in the hospital, mean differences did not reach significance for comparisons across the Behavioral Risk ($\bar{M}=14.2days$), Psychiatric and Substance Use Risk ($\bar{M}=14.5days$), and Cardiovascular Risk clusters ($\bar{M}=13.8days$; all *p* > 0.05), but did so for each of these clusters when compared with the Elevated Cardiovascular and Neuropsychiatric Risk cluster ($\bar{M}=11.3days$, *p*-values = 0.02, 0.005, and 0.05, respectively, all Cohen's d sizes = 0.2). Finally, although days in the ICU for the two younger groups (Behavioral Risk [$\bar{M}=8.3days$] and Psychiatric and Substance Use Risk [$\bar{M}=7.5days$]) do not differ significantly from one another (*p* = 1.0) or from the Cardiovascular Risk cluster ($\bar{M}=8.5days$, *p*-value = 1.0, *p*-value = 0.7); patients in all three clusters spent more time in the ICU in comparison with the Elevated Cardiovascular and Neuropsychiatric Risk cluster ($\bar{M}=$5.8 days, *p* − values < 0.001, 0.04, and < 0.001, *respectively*, all Cohen's d sizes = 0.2).

## Discussion

We aimed to understand relative frequency of distinct PECs after trauma and the influence PECs have for mortality and early outcomes indicators for msTBI. We leveraged the enormous resources in the PTOS to conduct a population-based study of PECs in TBI. Clusters of clinically relevant PECs can be reliably observed in trauma populations, and hold important implications for early patient outcomes. The data also reveal that the frequency for both simple PEC count (i.e., number of PECs any one patient may have) and PEC clusters (symptom groupings) are critically influenced by age and even injury severity, but their respective effects on post-injury outcomes may emerge months to years following hospital discharge, and thus are not captured in these hospital admission data.

### Impact of PECs on acute TBI recovery trajectories

Within the patients with msTBI who were examined, the sheer number and types of PECs had consequence for outcomes. First, as the number of PECs increases, mortality rate climbs incrementally by a factor of 1.7–1.9. Second, distinct PEC cluster types confer differential risk for recovery following injury, and perhaps unsurprisingly, age plays a critical role in the emergence of PECs (Fig. 5). In the early recovery period, these PEC clusters also confer risk of differential recovery following msTBI as measured by FIM, days spent in the hospital, and days in the ICU. The impact of PECs on recovery from TBI appears linked to the interactive characteristics of the chronic conditions carried by the patients with msTBI examined here, rather than the overall total number of PECs as evidenced by the small effect sizes associated with our results.

### Age and PECs hold association with injury severity

Given the preponderance of mTBI injuries (more than 60%) across all four clusters in the initial K-modes clustering results, we also suggest these data may speak to differences in injury mechanism as a result of both age and PEC types.^65^ More specifically, although the younger PEC profiles present with lower total number of PEC sub-categories, they also have a greater proportion of severe TBI cases in comparison with the two older PEC profiles, representing a trade-off between decreasing injury severity with age, and increased aggregation of age-dependent PECs. Further, only 6% or fewer of patients with TBI endorsed one PEC exclusively. More specifically, it appears that PEC diagnoses frequently co-occur, and this comorbidity does not appear restricted solely to a function of advanced age.

### Influence of sex on PECs

Patient sex proportions also appear to shift within the PEC clusters. Although the Behavioral Risk cluster contains the largest proportion of injured males, the Psychiatric and Substance Use Risk cluster demonstrates an increased number of injured females despite being of similar mean age and proportions of TBI severity. A key sex-by-PEC sub-category difference between these two clusters is the Psychiatric/Personality Disorder diagnosis; 43% of patients within this cluster endorsing this trait were female. Within the Cardiovascular Risk and Elevated Cardiovascular Risk clusters, an increase in mean age also emerges alongside a switch from a greater number of males to more female injuries, and an increase in the total number of reported PECs, aligning with prior research in both TBI and aging.^38^

### Inflammation and immunosenescence as part of TBI research

Accumulation of PECs induces necessary inflammatory processes in the acute phase; however, prolonged exposure to sub-clinical hyper-inflammation resulting from chronic conditions compromises the body's ability to effectively respond to additional physical and neurological insults.^66–69^ Despite established research examining reciprocal impacts between central and peripheral inflammatory processes,^19,41,42,70,71^ research protocols frequently detail omission of potential participants with diagnosed PECs, notably psychiatric conditions. As global populations continue to age, understanding the impact of multiple morbidity, and the interactions between timing and type of PEC onset, notably with advancing age, remain critical avenues for research determining both risk of and recovery from injuries.^30,72,73^

More specifically, overly restrictive exclusion criteria can eliminate the variability imparted by the presence of PECs, and impact understanding of health mechanisms that influence outcomes trajectory.^31,38,74,75^ For example, Isokourtti and colleagues^74^ demonstrated that in a Finnish cohort of patients with mTBI (*n* = 3023), only 2.5% (76 patients) met criteria for isolated mTBI, that is a patient with mTBI absent of any pre-existing medical or mental health problems. The broad PEC categories described by Isokourtti and colleagues^74^ are consistent with both our findings here and mirror research in TBI and non-TBI patient cohorts across the lifespan.^15,30,31,35,76,77^ Patients with msTBI presenting with no diagnosed PECs comprise only about 25% of the TBI sample analyzed here, so continued focus on “isolated TBI” samples for research protocols will not only exclude a majority of affected patients with TBI (thus leading to poor generalizability of findings), but also eliminate opportunities to examine the comorbidities complicating patient recovery and the associated costs. Further, TBI research is under-served by the under-representation of geriatric patient samples,^38,78–80^ often due to restrictive inclusion criteria.^38,81,82^ This poses significant challenges to understanding post-injury outcomes that may emerge years to decades post-injury.^83,84^ This is particularly concerning, given the increase in TBI-related hospitalizations and deaths in geriatric populations in comparison with other age demographics over the last several years.^38,65,78,80,81^

Our findings align with a growing literature examining the impact of pre-existing health conditions on recovery following TBI. More specifically, Kumar and colleagues^15^ implemented Treelet Transform classification, which resulted in three clusters, which they broadly classified as “acute medical diseases/infections, chronic conditions, and substance abuse disorders.” Similarities between our results and the Treelet Transform clusters offered by Kumar and colleagues include the emergence of chronic conditions, specifically those impacting cardiovascular health, such as cardiac conditions (including heart disease) and diabetes. Additional commonalities include the emergence of substance use disorders, a prominent feature in two of our four clusters. Two important distinctions between our classification methods, however, warrant mention. First, our resulting clusters from K-modes clustering do not permit patients to belong to more than one cluster, whereas Treelet Transform clustering undertaken by Kumar and colleagues^27^ does permit patients to have overlapping cluster membership. Second, the PTOS defines PECs as conditions present before admission to the hospital, and thereby does not code for hospital-acquired infections (nosocomial conditions); this is in contrast to the data analyzed by Kumar and colleagues,^15^ which included hospital codes documenting health conditions present prior to hospital entry and those acquired during patients' hospital stays.

Further, one of our overarching research aims of descriptively examining all PECs rather than focusing on an individual or subset of conditions aligns with yet additional work by Kumar and colleagues.^28^ Although our work presented here stems from trauma center data in the acute stage post-injury, and Kumar and colleagues^28^ examined outcomes 10 years post-injury, our findings speak to the importance of studying multi-morbidity as it better simulates real-world conditions. Further, Kumar and colleagues^28^ also argue that physical and mental health conditions may reciprocally exacerbate one another, a qualitative aspect that is illustrated within our K-modes clustering results, and is highlighted in the discussion offered in this article regarding exclusion criteria that prohibit individuals with these conditions from enrolling in research.

### Limitations and future directions

The results and proposed clinical implications should be considered in the context of the following caveats. The hospital-based data analyzed here may be impacted by an inherent selection bias of individuals who are more likely to seek medical services. Similarly, the de-identified nature of the data prevented assessment of whether the cases analyzed here were repetitive admissions for trauma. Repetitive experiences of TBI, and both neurological and psychiatric conditions have been demonstrated to confer greater risk of hospital re-entry for TBI.^29^ Additionally, given the emergency/trauma center environment, the cases analyzed here do not have pre-injury functional status data available to assess change in functional status from before hospital admission. Further, the cross-sectional data here do not delineate when patients were diagnosed with PECs, precluding our ability establish direct causality of specific PECs on either chronological acquisition of systemically related PECs or injury specifically.^83^

Relatedly, the availability of insurance (and access to care) may also impact whether PECs can be diagnosed, subsequently disclosed by these patients when they present for care, and may impact inclusion in research examining the questions presented here. The PTOS data permit coding for an array of insurance types,^60^ ranging from those without insurance coverage to those with a private indemnity, and the data presented here are from patients from every possible category of insurance (6.8% did not identify a third-party payor and bills for service were rendered to the patient, compared with 7.9% prior to data cleaning). As a final point, prior work in our lab demonstrates that these differences in insurance exert influence on post-discharge outcomes, rather than the outcomes linked to acute hospital stay presented here.^85^

Further, the proportion of African-American patients without recorded PEC data, and the degree to which Hispanic ethnicity was missing from the data make it difficult to generalize these analyses to non-Caucasian patient populations. This data gap remains both concerning and critically important, considering current statistics that non-Caucasian patients are more likely to suffer from a greater number of PECs, have a higher incidence of TBI, and are at greater risk for developing dementia-related conditions (for which both prior TBI and pre-existing conditions confer greater risk).^86^ Given the known impact of race and ethnicity status on TBI outcomes, neurotrauma investigators should be proactive about how to guarantee that aggregated data are representative of brain injury as it occurs in the population, and that includes all race and socioeconomic demographics.

## Conclusion

The findings presented across the aims of this study demonstrate that implementing a data-driven approach revealed distinct groups (clusters) of PECs across the lifespan. The prominent PECs present in these four clusters emerge at specific ages, with increasing age associated with a simultaneous increase in the quantity of specific age-related conditions, but also decreasing TBI severity. Similarly, PEC quantity and type were significantly, yet weakly associated with measures of functional independence and hospital stay. These findings add to the literature examining the impact of pre-existing conditions on risk of and recovery from TBI, and infer the importance of re-conceptualizing the impact of PECs on recovery above and beyond a single number or solitary condition. Continued inclusion of individuals absent any diagnosed PECs in TBI research captures only a limited scope of the population, indirectly omitting older and non-Caucasian patients with TBI who may be at greater risk for exacerbated recovery or pathological aging, and presents salient challenges to understanding recovery mechanisms post-TBI as well as for reproducibility of findings.

## Supplementary Material

Supplemental data

## Abbreviations Used

ADHD: attention deficit hyperactivity disorder
ANOVA: analysis of variance
CDC: Centers for Disease Control and Prevention
COPD: chronic obstructive pulmonary disease
CVD: cardiovascular disease
ED: emergency department
FIM: functional independence
GCS: Glasgow Coma Scale
ICD9: *International Classification of Diseases*, 9th Revision
ICU: intensive care unit
LTC: long term care facility;
msTBI: moderate-to-severe TBI
mTBI: mild TBI
NCCDPHP: National Center for Chronic Disease Prevention and Health Promotion
NTDB: National Trauma Data Bank
OR: odds ratio
PEC: pre-existing health conditions
PTOS: Pennsylvania Trauma Outcomes Study
PTSF: Pennsylvania Trauma Systems Foundation
SD: standard deviation
TBI: traumatic brain injury
wss: within-cluster sum of squares
