# Supplementary material for: A Population-Based Study of Pre-Existing Health Conditions in Traumatic Brain Injury
Source: Neurotrauma Rep. 2021 Jun 9;2(1):255–69. doi: 10.1089/neur.2020.0065 (PMC8244518; doi:10.1089/neur.2020.0065)
Supplement: Supplemental data [file Supp_TableS1.docx]

| Supplementary Table 1: PEC parent codes and subcategory definitions as defined by the PTSF | |
| --- | --- |
| Code | Designation |
| **A** | **Cardiac Disease** |
| A.01 | History of cardiac surgery |
| A.04 | Cor pulmonale |
| A.02 | Coronary artery disease |
| A.03 | Congestive heart failure |
| A.05 | Myocardial infarction |
| A.06 | Hypertension requiring medication |
| A.07 | Congenital Cardiac Disease |
| **B** | **Diabetes** |
| B.01 | Insulin-dependent diabetes mellitus |
| B.02 | Non-insulin dependent/Type II Diabetes |
| B.03 | Diabetes Mellitus – collapses previous two codes B.01 and B.02 |
| **C** | **Disease. *We elected to call this Gastric Diseases/Conditions** |
| C.03 | Pancreatitis |
| C.04 | Inflammatory bowel disease |
| C.01 | Peptic ulcer disease |
| C.02 | Gastric/esophageal varices |
| C.05 | Bariatric Surgery |
| **D** | **Hematologic Disorders** |
| D.01 | Acquired coagulopathy |
| D.04 | Pre-existing anemia |
| D.03 | Hemophilia/Clotting Disorders |
| D.02 | Reversible Anticoagulant Therapy |
| D.05 | Anti-platelet Agents |
| D.06 | Thrombocytopenia/Platelet Disorders |
| D.07 | Pradaxa Therapy |
| D.08 | Other Bleeding Disorder |
| D.09 | Chronic Aspirin Use |
| D.10 | Anticoagulant Therapy |
| **E** | **History of psychiatric disorders** |
| E.00 | History of psychiatric disorders, mental/personality disorder |
| E.01 | Attention Deficit Disorder (ADD) |
| E.02 | Intellectual Disability |
| **F** | **Immunosuppressed** |
| F.01 | HIV/AIDS |
| F.02 | Routine Steroid Therapy |
| F.03 | Transplants |
| F.04 | Active chemotherapy |
| **G** | **Liver Disease** |
| G.01 | Bilirubin > 2 mg % (on admission) |
| G.02 | Documented history of cirrhosis |
| **H** | **Malignancy** |
| H.01 | Undergoing current therapy |
| H.02 | Current or existence of metastasis |
| H.03 | History of Pediatric Malignancy |
| **I** | **Musculoskeletal** |
| I.01 | Arthritis |
| I.02 | Systemic lupus erythematosus |
| I.03 | Osteogenesis |
| **J** | **Neurologic** |
| J.07 | Organic brain syndrome |
| J.08 | Parkinson’s disease |
| J.02 | Multiple sclerosis- beginning in 2013 used in place of code J.05 |
| J.04 | Seizures |
| J.05 | Chronic demyelinating disease |
| J.03 | Alzheimer’s disease |
| J.06 | Chronic dementia |
| J.01 | Spinal cord injury |
| J.09 | CVA/Hemiparesis (stroke with residual) |
| J.10 | Autism Spectrum |
| J.11 | Palsy (CP) |
| J.12 | Dementia- beginning in 2017, collapses prior separate codes J.03 and J.06 |
| **K** | **Obesity** |
| K.00 | Obesity |
| **L** | **Pulmonary Disease** |
| L.01 | Documented prior history with ongoing active treatment |
| L.02 | Asthma |
| L.03 | Chronic obstructive pulmonary disease |
| L.04 | Chronic pulmonary condition |
| L.05 | Respiratory Disease Chronic Obstructive Pulmonary Disease (COPD)- collapses above 4 codes |
| **M** | **Renal Disease** |
| M.01 | Serum creatinine > 2 mg % (on admission) |
| M.02 | Dialysis (excludes transplant patients) |
| **N** | **Substance Abuse** |
| N.01 | Drug Use Disorder Substance Abuse Disorder |
| N.02 | Chronic alcohol abuse |
| **P** | **Pregnancy** |
| P.00 | Pregnancy |
| **Q** | **Any Previous History of Admission for Trauma or Burn (regardless of facility)** |
| Q.00 | Any Previous History of Admission for Trauma or Burn |
| Q.01 | Previous Head Trauma |
| **R** | **Endocrine** |
| R.01 | Thyroid Disease |
| **S** | ***No parent category listed in guide doc from PTSF. We elected to call this "Vascular Risk".** |
| S.01 | Ascites within 30 days |
| S.02 | Current Smoker |
| S.03 | Advanced Directive Limited Care |
| S.04 | Functionally Dependent Health Status |
| S.05 | History of angina within past 30 days |
| S.06 | History of PVD Peripheral Arterial Disease |
| S.07 | Prematurity |
| S.08 | Pre-hospital cardiac arrest with CPR |
| S.09 | Angina Pectoris |
| **T.00** | **Congenital Disorder (Specify)** |
